# Medical and Physical Intelligence

**Published:** 1800-09

**Authors:** 


### MEDICAL AND PHYSICAL INTELLIGENCE.

### *Prof. Abildgaard on Refpiration.*

PROFESSOR ABILDGAARD, of Copenhagen, has made feveral experiments on refpiration, the refults of which feem to be quite in oppofition to the generally prevailing theories on this fubject. Although they are not fo perfect and extenfive as to fubvert entirely the common theory, yet they deferve the attention of every phyfiologift, and may contribute to a re-examination of feveral phænomina of refpiration, which have been hitherto, by no means, fatisfactorily explained. 1. It has, in general, not been fufficiently regarded how fmall the quantity of air is infpired at every breath, which, according to his experiments, does not exceed three cubic inches, a portion of air only fufficient to fill the wind pipe (arteria afpera). It appears, therefore, that at every refpiration the air contained in the arteria afpera is only renewed, but that the air in the veficles and cells of the lungs is accordingly very flowly replaced by frefh air. However, a want of breath, or renewed air, only lafting a few minutes, is able to kill animals, which does not feem to correfpond with this phenomenon. 2. Experience fhews that the retention of breath caufes the fame uneafinefs and inconveniencies after infpiration as after expiration; though, according to theory, it fhould be eafier to retain the breath after infpiration. 3. The wind-pipe was opened in two dogs, and by a convenient apparatus, azotic gas injected into the lungs of one, and oxygen gas into thofe of the other; and after the lungs feemed thereby perfectly extended, a ligature was applied to their wind-pipes. Both dogs felt the fame painful fenfation in the attempt of gafping for breath, and there was no difference perceived except that the latter lived eleven minutes, whereas the former died in five. The movement of the heart ftill remained for fome time vifible in both after the breathing had ceafed. The queftion now is, why both animals attempted to breathe with equal fenfation of pain and uneafinefs, though one had the moft falubrious air in the lungs? and why did they die before the movement of the heart, and confequently the circulation had ceafed? 4. It has been obferved by repeated experiments, that animals being in a ftate of afphyxia, and brought to life again by air being injected into their lungs, did not catch breath before the heart began to beat; if, therefore, the motion of the heart is fuppofed to ceafe from want of oxygen, why does it beat in this cafe, before breathing is re-eftablifhed? 5. Air drawn from the lungs of a dog killed by a ligature of the wind-pipe, was found to contain a portion of oxygen, and yet the dog died for want of breath, which feems contrary contrary to the common opinion of the ufe of oxygen, &c. Prof. Plaff, of Kiel, remarks, in oppofition to thefe experiments, that No. 1, is inconfiftent with other experiments made on this fubject by Robert Menzies (in his Tentamen Phyfiologicum de Refpiratione) with the greateft exactnefs, as it feems, according to him, forty-three cubic inches are always infpired at once. They do not likewife correfpond with the experiments of Lavoifier, Abernethy, and Goodwyn, as, according to the latter, about ten cubic inches of vital air, (oxygen gas,) and nearly forty cubic inches of atmofpheric air, are breathed in at every refpiration. The difference between thefe experiments and Prof. Abildgaard's is confequently too great, and fome inaccuracies muft have been committed on one fide or the other. It is therefore to be wifhed, that Prof. A would relate his experiments at large, to enable us to judge to which of them credit is to be given. The obfervation in No. 3, is indeed interefting, but could not the fame painful gafping for breath in the dog, whofe lungs were filled with oxygen gas, be derived from the preternatural extenfion of them, and the mechanical impediment caufed thereby in the circulation of the blood? the ligature itfelf may have had fome influence here. Befides, it is probable that the carbonic acid evolving itfelf in the lungs extended by oxygen gas, may have produced the fame phenomena in this dog as the mephitic air in the other, particularly as we know that a mixture of air, in which oxygen gas is prefent, may become fatal by an additional quantity of carbonic acid; and this likewife explains why the animal (No. 5) died, though the air in its lungs contained a portion of oxygen gas. The previous motion of the heart (No. 4) before refpiration was perceived, may, notwithftanding, be owing to the oxygen entering the lungs with the injected air, which ftimulated the heart previous to the re-eftablifhment of refpiration. *(See Northern Archives of Medical and Natural Science, by Prof. Plaff and Dr. Scheel, Vol. I. No. 1. in German.)*

---

### *Method of obtaining Sugar and Brandy from the Beet Root.*

The committee for examining Mr. Achard's mode of fabricating fugar, confifting of the moft refpectable chemifts at Berlin, has now, by order of the King of Pruffia, publifhed an inftruction, containing the method of making that indigenous fugar, of which the following is the moft effential:—The beet roots beft calculated for the extraction of fugar, are thofe which have a foft flefh, whitifh towards the edges, and not growing above ground. After being cleaned, they are boiled, cut into pieces, and pounded in a wooden trough with wooden ftampers, and afterwards preffed. The juice thus obtained is immediately put into a polifhed copper kettle and fimmered, during which time the fcum appearing on its furface muft continually be taken off. To one hundred quarts of this juice, two ounces or lefs of flacked lime, diluted fo as to have the appearance of milk, are added, and the boiling continued till the juice is thickened to the half of it. Having ftrained it through a woollen a woollen cloth, it is thickened in a polifhed copper veffel, or, which is better, in a tin veffel, to the confiftency of a fyrup, which afterwards is put into glafs, ftone, or wooden veffels. Thefe being placed near a moderate fire, faccharine cryftals appear, which being freed by expreffion from the mucilaginous juice, a very good raw fugar is obtained, which muft now be left to the refiner.

For the preparation of brandy, the water ufed in the firft boiling of the roots, is boiled again, and poured on the refiduum from the firft expreffion of the pounded roots; this muft ftand for a day or two, after which it is expreffed, and the remaining dry pulp ferves as a good food for cattle. The juice obtained in this way is mixed with the wafte parts of the fyrup and the mucilage which remains after the expreffion of the faccharine cryftals, and all boiled together till half of it is evaporated. The liquor is then poured into a coop expofed to a temperature of 45° Fahrenheit, and cooled to 65°. Having added a proportionate quantity of yeaft, it is left to ferment, and in three or four days after the diftillation is undertaken. The tafte and fmell of turnips are removed by the addition of well-burned pulverifed charcoal; but the brandy thus obtained has a great deal of the flavour of rum. According to the experiments made by the Committee, 15 quintals of beet-root afforded 57½ lb. of raw fugar and near five gallons of brandy.

---

### *We have authority to announce the following Medical Lectures.*

### St. Thomas's and Guy's Hospitals.

The Lectures for the enfuing Seafon will commence at thefe united Hofpitals in the following order:—

*Theatre, St. Thomas's Hofpital.*

Anatomy and Surgery, on Wednefday, October 1, at one o'clock, by Mr. Cline and Mr. Astley Cooper.

*Theatre, Guy's Hofpital.*

Theory and Practice of Medicine, on Friday, October 3, at ten in the morning, by Dr. Babington.—Midwifery, and Difeafes of Women and Children, on Saturday, October 4, at eight in the morning, by Dr. Lowder and Dr. Haighton.—Chemiftry and Experimental Philofophy, on the fame morning at ten, by Dr. Babington and the Rev. Mr. Roberts.—Phyfiology, or Laws of the Animal Economy, on Monday, October 6, at a quarter before feven in the evening, by Dr. Haighton.—Therapeutics and Materia Medica, on Thurfday, October 9, at feven in the evening, by Dr. Curry.—Principles and Practice of Surgery, on Monday, October 13, at eight in the evening, by Mr. Astley Cooper.——Mr. Cooper will alfo deliver a Courfe of Lectures on Select Surgical Cafes in the Hofpital.

In addition to thefe, early in October, a Courfe of Clinical Lectures will be begun by Dr. Saunders and Dr. Babington, and continued through the Seafon on the following plan:

The

The moſt interesting Caſes will be ſelected from among the whole number of Medical Patients admitted into the Hoſpital.

As ſoon as poſſible after admiſſion, a Hiſtory of the Caſe will be taken down at length; in which will be deſcribed the Patient's age, temperament, occupation, and condition in life; the date and progreſs of the complaint; the actual or probable cauſes giving riſe to it; the medicines, if any, already employed, and the effects they have had; together with the ſymptoms preſent, whether collected from the Patient's feelings, or cognizable by the Phyſician. There will alſo be given a daily Report, ſpecifying the remedies preſcribed, their ſenſible effects, and the changes which take place in the diſeaſe. Every Thurſday evening, at eight o'clock, a Lecture will be delivered upon the moſt important caſes; in which the ſymptoms of each will be examined, the leading and eſſential ones pointed out, and the diſeaſe referred to its proper place in the beſt Syſtems of Noſology. A comprehenſive view will be taken of the plan of cure to be followed in ſuch caſes; the ſeveral indications will be laid down; and the particular circumſtances, either originally belonging to the caſe, or ariſing during its progreſs, and which may render a deviation from the general plan neceſſary, will be ſpecified. The ordinary and expected effects of the remedies preſcribed will be mentioned; their operation diſtinguiſhed from the conſequences of the complaint; and any new or unuſual reſult from them will be carefully noted, and, as far may be, accounted for. The ſymptoms that portend a favourable event, or the contrary, will be marked as they occur, and connected with the previous circumſtances, in order to aſcertain the dependance of the one upon the other, and afford data upon which to g ound an early and correct prognoſtic. Laſtly, where the termination is fatal, and the caſe is ſuch as to be elucidated by examination of the body, this will, if poſſible, be effected, and a full account given of the morbid appearances. In ſhort, throughout the whole Courſe, the great object will be to unite Theory with Practice, to verify general precepts by individual examples, and thereby to give a connected and ſyſtematic view of the Hiſtory, Nature, Cauſes, and Cure of Diſeaſe.

### CONDITIONS.

1. Clinical Pupils to pay five guineas for the Courſe, which will commence in October, and end in May; the firſt four months to be conducted by Dr. Saunders, the next by Dr. Babington.

2. No perſon to be accepted as a Clinical Pupil, who is not intitled to attend the Lectures on the Practice of Medicine during the time the Clinical Courſe will continue.

3. Clinical Pupils to have the privilege of going into the Clinical Wards only, unleſs they are likewiſe Pupils either to the Phyſicians or Surgeons of the Hoſpital; and then only during Hoſpital hours.

4. Gentlemen entering to the Phyſicians as perpetual Pupils, or for twelve months, to have the benefit of the Clinical Lecture without additional expence.

TERMS of attending as PHYSICIANS PUPILS.

For six months, 10 guineas.—For twelve months, 15 guineas.—Perpetual, 21 guineas.

---

The following Lectures will commence at the London Hospital on the 1st of October: On the Theory and Practice of Physic, by Dr. COOKE; on Chemistry, by Drs. HAMILTON and FRAMPTON. The Clinical Lectures, by Drs. COOKE, HAMILTON, and FRAMPTON. The Anatomy, Physiology, and the Principles and Operations of Surgery, by Mr. BLIZARD and Mr. T. BLIZARD. The Anatomical Demonstrations by Mr. HEADINGTON.

---

THEATRE of ANATOMY, Great Windmill Street.

Mr. WILSON will commence his Course of Lectures on Anatomy, Physiology, Pathology, and Surgery, on Wednesday, the 1st of October, at two o'clock.

A Room is likewise open for Dissections, under the direction of Mr. WILSON and Mr. THOMAS, from nine o'clock in the morning till two in the afternoon, from the 10th day of October till the 20th of April; where regular and full demonstrations of the parts dissected are given; where the different cases in Surgery are explained, the methods of operating shewn on the dead body; and where also the various arts of injecting and making Preparations are taught.

---

Dr. FORDYCE will commence his Autumnal Course of Lectures on the Practice of Physic, Materia Medica, and Chemistry, at his house in Essex-street, Strand, on Monday the 6th of October, at eight o'clock in the morning.

---

Dr. PEARSON's Lectures on Physic and Chemistry will commence the first week in October next, at the Laboratory in Whitcomb-street, Leicester-square. The Lectures on the Materia Medica and Practice of Physic are delivered every morning, from a quarter before eight to a quarter after nine, and on Chemistry from a quarter after nine to ten. Lectures on the Cases of Hospital Patients are also given once a week.

---

Dr. CRICHTON will commence his usual Autumnal Course of Lectures on the Theory and Practice of Physic, Chemistry, and Materia Medica, on Monday, October 13, at his house in Clifford-street, Bond-street, at eight and nine o'clock in the morning.

---

Dr. BRADLEY will commence his Autumnal Course of Lectures on the Theory and Practice of Medicine, on Monday, October 6, at the Lecture Room, No. 102, Leadenhall-street, at six o'clock in the afternoon.

---

Dr. OSBORN's and Dr. CLARKE's Lectures on Midwifery and the Diseases of Women and Children will be given the following winter, winter, as ufual, only at Dr. Clarke's houfe, No. 1, in New Burlington-ftreet. The Courfe will begin on the firft Monday in October next, at half paft ten o'clock.

---

Dr. Dennison and Dr. Squire, on Wednefday, Oct. 1, will begin a Courfe of Lectures on the Principles and Practice of Midwifery, and the Difeafes of Women and Children. In addition to a conftant fupply of Labours, Gentlemen may have frequent opportunities of vifiting patients in diforders during pregnancy, and after delivery.

---

Dr. Batty will begin his Courfe of Lectures on the Theory and Practice of Midwifery, and on the Difeafes of Women and Children, on Monday, October 6, at his houfe, No. 6, in Great Marlborough-ftreet, at eleven o'clock in the morning.

---

On the ninth day of the enfuing month, October, T. Pole, Man-Midwife to the Obftetric Charity for the Delivery of poor Women at their own Habitations, will commence his Autumnal Courfe of Lectures on the Theory and Practice of Midwifery, including the Difeafes of Women and Children, at his houfe, No. 102, Leadenhall-ftreet, near the Royal Exchange, London.

---

Mr. Pearson will commence his Autumnal Courfe of Lectures on Surgery, at his houfe in Golden Square, on Monday, Oct. 6, at feven o'clock in the evening.

---

Mr. Chevalier's Autumnal Courfe of Lectures on the Principles and Operations of Surgery, will commence on Monday, October 6, at his houfe, No. 20, South Audley Street. The terms of attendance are Three Guineas.

---

Mr. Carpue will continue his private Anatomical Lectures, at his houfe, No. 44, Leicefter-fquare. Particulars may be known by applying to Mr. C.

---

Meffrs. A. and C. R. Aikin will refume their courfe of Lectures on Chemiftry and the Chemical Arts, early in the winter, at the houfe of Mr. C. R. Aikin, Surgeon, Broad Street Buildings.

---

In the prefs, an Effay on Phlegmatia Dolens, or the painful Swelling of the lower Extremities incident to Lying-in Women. In which a literary hiftory of this difeafe, comprehending a review of the different theories that have been formed concerning it, from the earlieft records of medicine to the prefent time, will be given; and its analogy with peritonitis puerperalis, or child-bed fever, will be pointed out and elucidated by a comparifon of their fymptoms, terminations, predifpofing, remote and proximate caufes, and their cure. By John Hull, M. D.

The

The fame Author has prepared for the prefs, an Epitome of the Nofologia Methodica of Sauvages, which is intended to be publifhed by fubfcription. The propofals and a profpectus of the work will appear in a few weeks.

---

A fecond edition is in the Prefs, and will be fpeedily publifhed, of Dr. CHISHOLM's Effay on the Malignant and Peftilential Fever of Grenada, greatly enlarged; comprehending additional facts and illuftrations in fupport of its nature and treatment, as formerly defcribed; with obfervations on contagion, the endemial fevers of the Weft Indies, the effects of the nitrous acid and oxygenated muriate of pot-afh in feveral difeafes, &c.

